# Graphene-enhanced visible-light photocatalysis of large-sized CdS particles for wastewater treatment

**DOI:** 10.1186/1556-276X-9-148

**Published:** 2014-03-26

**Authors:** Wei Lü, Jie Chen, Yao Wu, Lianfeng Duan, Yue Yang, Xin Ge

**Affiliations:** 1 Key Laboratory of Advanced Structural Materials, Ministry of Education, Changchun University of Technology, Changchun 130012, China; 2 Advanced Institute of Materials Science, Changchun University of Technology, Changchun 130012, China; 3 State Key Laboratory of Rare Earth Resource Utilization, Changchun Institute of Applied Chemistry, Chinese Academy of Sciences, Changchun 130022, People's Republic of China

**Keywords:** CdS, Graphene, Photocatalysis, Wastewater treatment

## Abstract

The hybrid composites of graphene decorated by large-sized CdS particles (G/M-CdS) were prepared by a one-pot solvothermal route in which the reduction of graphite oxide into graphene was accompanied by the generation of microsized CdS particles. The structure and composition of the obtained nanocomposites were studied by means of X-ray diffraction, scanning electron microscopy, and transmission electron microscopy. The CdS particles with the average sizes of approximately 640 nm were formed on graphene sheets. The as-prepared composite was used as adsorbent to remove dye from wastewater using the organic dye Rhodamine B as the adsorbate. The G/M-CdS composite reveals a high photodegradation rate under visible light irradiation. Our results demonstrate that the G/M-CdS is very promising for removing organic dyes from wastewater.

## Background

In recent years, the growing concerns about energy and environmental problems have stimulated extensive research on solar energy utilization. It is well known that dyes are widely used in various fields, but their discharge into water could cause environmental pollutions since most of the dyes are harmful. Therefore, various strategies are explored to photocatalytic degradation of organic dyes using semiconductor photocatalysts. In particular, the carbon nanostructures, acting as outstanding electron acceptors and highly conductive scaffolds, have found their applications in photocatalysis [1-4]. Commonly used adsorbents can suffer from low adsorption capacities and separation inconveniences. Therefore, the exploration of new promising adsorbents is still desirable.

Graphene with atomically thin and two-dimensional conjugated structure, exhibits high conductivity as well as thermal, chemical, mechanical, and optical stability and a high specific surface area [5-8]. These outstanding advantages allowed graphene to be utilized as a promising adsorbent supporting material to remove pollutants from aqueous solution [9-14]. CdS is an important II–VI semiconductor, it can be potentially applied in many fields such as light-emitting diodes, thin film transistors, solar cells, and photocatalysts [15-19]. The narrower bandgap of CdS than that of TiO_2_ facilitates the utilization of visible light, which makes CdS a competitive candidate as photocatalyst. When CdS is irradiated by visible light, electrons located in the valence band can be excited to the conduction band, forming electron-hole pairs, which are responsible for the photocatalytic activity. Disadvantageously, the rapid recombination of the excited electron-hole pairs is an obstacle limiting the photocatalytic activity of catalysts. The ways to delay the electron-hole pair recombination of CdS include the hybrid of CdS with other semiconductors [20,21], noble metals [22], or loaded CdS on support materials with high surface areas [23] or combining CdS with conductive supports [24]. The nanocomposites composed of CdS and graphene showed significantly improved properties in electrocatalysis, supercapacitor, high-performance lithium ion batteries, etc. As for graphene-based composite photocatalysts, the *π-π* conjugation net and the conductivity made graphene an efficient electron acceptor, when the semiconductors were excited, the electrons at the interface could be transferred to graphene and stabilized by the conjugation net, retarding the charge recombination. The applications of graphene-CdS nanocomposites as the adsorbent for the extraction of organic pollutants have been reported [25-30]. The above methods share one common feature: nanoscaled CdS nanocrystals were attached onto the surface of graphene. Very recently, Wang et al. reported the photocatalysis investigation using nest-like CdS-graphene composite, and the nest-like CdS structure with an average diameter of about 1 μm is composed of many branches with approximately 5-nm diameter [31]. It is well known that the size of inorganic materials have a large influence on their widely changing chemical or physical properties. However, the photocatalysis properties of CdS microparticles-graphene composites (G/M-CdS) have not been really reported previously.

Herein, we synthesized the G/M-CdS composites by one-step hydrothermal method. Its practical application potential in the removal of dyes from aqueous solution was investigated. As indicated previously, organic dyes are widely used in various fields, which are the main organic pollutant source in water. These dyes own the same feature on structure in that benzene rings are included. Therefore, in order to evaluate the adsorption performance and photocatalytic activity of the G/M-CdS, one representative organic dye including benzene rings should be chosen. Rhodamine B (Rh.B) is a chemical compound and a typical dye, which is often used as a tracer dye within water and is used extensively in biotechnology applications. Thus, Rh.B was selected as model organic pollutant in this work. The results exhibit that the G/M-CdS composites possesses very efficient adsorption and photodegradation ability. To the best of our knowledge, this is the first attempt to treat wastewater with large CdS particle/graphene composites.

## Methods

All the chemicals and reagents were of analytical purity and used without further purifications. CdCl_2_ · 2.5H_2_O, Na_2_S_2_O_3_ · 5H_2_O and Rh.B were purchased from Aladdin. Water used in all experiments was doubly distilled and purified by a Milli-Qsystem (Billerica, MA, USA). Transmission electron microscopy (TEM) images were obtained using a JEOL2010 transmission electron microscope (Akishima-shi, Japan). The powder X-ray diffraction (XRD) measurements were performed using a D-MAXIIA X-ray diffractometer (Rigaku, Shibuya-ku, Japan) with CuKa radiation (*λ* = 1.5406 Å). The concentrations of dye solutions were measured using a UV-2501 spectrophotometer (Shimadzu, Kyoto, Japan).

Graphite oxide (GO) was synthesized from natural graphite powder (spectral requirement, Shanghai Chemicals, Shanghai, China) according to a modified Hummers method. The G/M-CdS composite was prepared according to previous reports [32,33]. Typically, 9 mg of GO was dispersed in 30 mL of deionized water by ultrasonication for 1 h. Then 1.5 mmol CdCl_2_ · 2.5H_2_O was added followed by 30-min stirring. Subsequently, 1.5 mmol Na_2_S_2_O_3_ · 5H_2_O was added. After 15-min stirring, the solution was transferred into a Teflon-lined stainless steel autoclave (50 mL) and reacted under 160°C for 10 h. After cooling to room temperature, the obtained solution was then centrifuged and washed by deionized water several times. Finally, the formed G/M-CdS composites were dried in a vacuum drier. For comparison, CdS microparticles (MPs) were also synthesized under the same reaction condition without adding GO.

Adsorption experiments were carried out in the dark. Rh.B was selected as an adsorbate, and G/M-CdS were used as adsorbents. A comparative test was also performed on CdS MPs. In every test, a known amount of G/M-CdS composite or CdS particles was added to 20 mL of dye solutions with the concentration 0.01 mg/mL. After reaching equilibrium, the suspension was centrifuged, and solution was analyzed for the concentration of Rh.B left using a spectrophotometer at *λ*_max_ = 554 nm. The removed quantity (*q*_eq_ in mg/L) of the dye by G/M-CdS could be calculated as

(1) $$q_{\text{eq}}=\frac{C_{0}-C_{\text{eq}}}{m}V$$

where *C*_0_ (mg/L) represents the initial dye concentration, *C*_eq_ (mg/L) is the equilibrium concentration of the dye remaining in the solution every test, *V* (L) is the volume of the aqueous solution, and *m* (g) is the weight of the G/M-CdS composite.

Photocatalytic experiments were conducted to photocatalytically degrade Rh.B in water under visible light irradiation. A domestic visible light lamp (11 W) was used as a light source and set about 10 cm from the reactor. Experiments were carried out at ambient temperature. The reaction suspension was prepared in the same fashion as in the adsorption experiments. Before irradiation, the solutions were stirred in the dark in order to reach the adsorption-desorption equilibrium. At different irradiation time intervals, analytical samples were taken from the reaction suspension and centrifuged to remove the photocatalyst particles. The concentrations of the remnant Rh.B were monitored by checking the absorbance of solutions.

## Results and discussion

**Figure 1:**
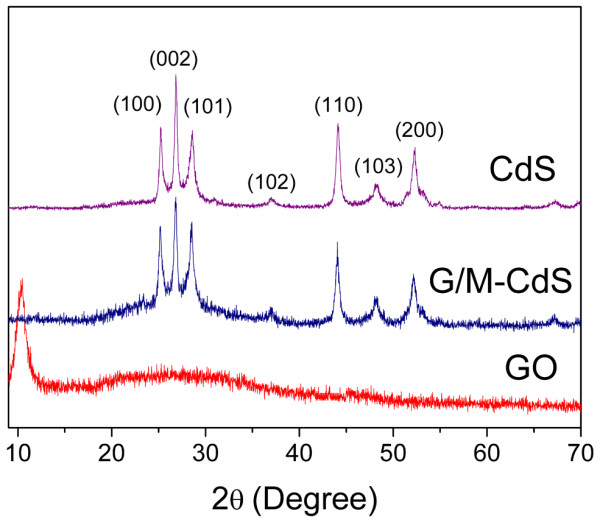
XRD patterns of the as-prepared CdS MPs, G/M-CdS, and GO samples.

As shown in Figure 1, XRD measurements were performed to obtain crystalline structural information for the as-synthesized GO, CdS MPs, and G/M-CdS. The GO presents a very sharp diffraction peak at 10.3°, whereas the weak and broad peak between 20° to 30° suggests residual unoxidized graphite. The characteristic peaks at 24.86°, 26.48°, 28.32°, 36.72°, 43.77°, 47.98°, and 52.0° correspond to (100), (002), (101), (102), (110), (103), and (200) planes of hexagonal-phase CdS crystals. The XRD results clearly suggest that the addition of graphene oxide did not influence the crystal structure of hexagonal phase CdS. The crystallinity of the G/M-CdS sample is very close to that of CdS, indicating that the GO supplies a platform in which the CdS particles can nucleate and grow. In addition, the 2*θ* degree of the peaks in pure G/M-CdS shifted a little to smaller coordinate numbers compared with those in pure CdS, which implies that the interplanar distance of graphene-coated CdS is larger than that of pure CdS. A possible reason to this might be that graphene nanosheets afforded electrons to Cd atom, which reduced the electrostatic attraction between Cd atom and S atom, and weakened the binding energy [34]. This phenomenon suggests that the G/M-CdS hybrid is formed. This result also agrees with previous works, in which GO is used as a support material to prepare graphene-based nanomaterials [35,36].

**Figure 2:**
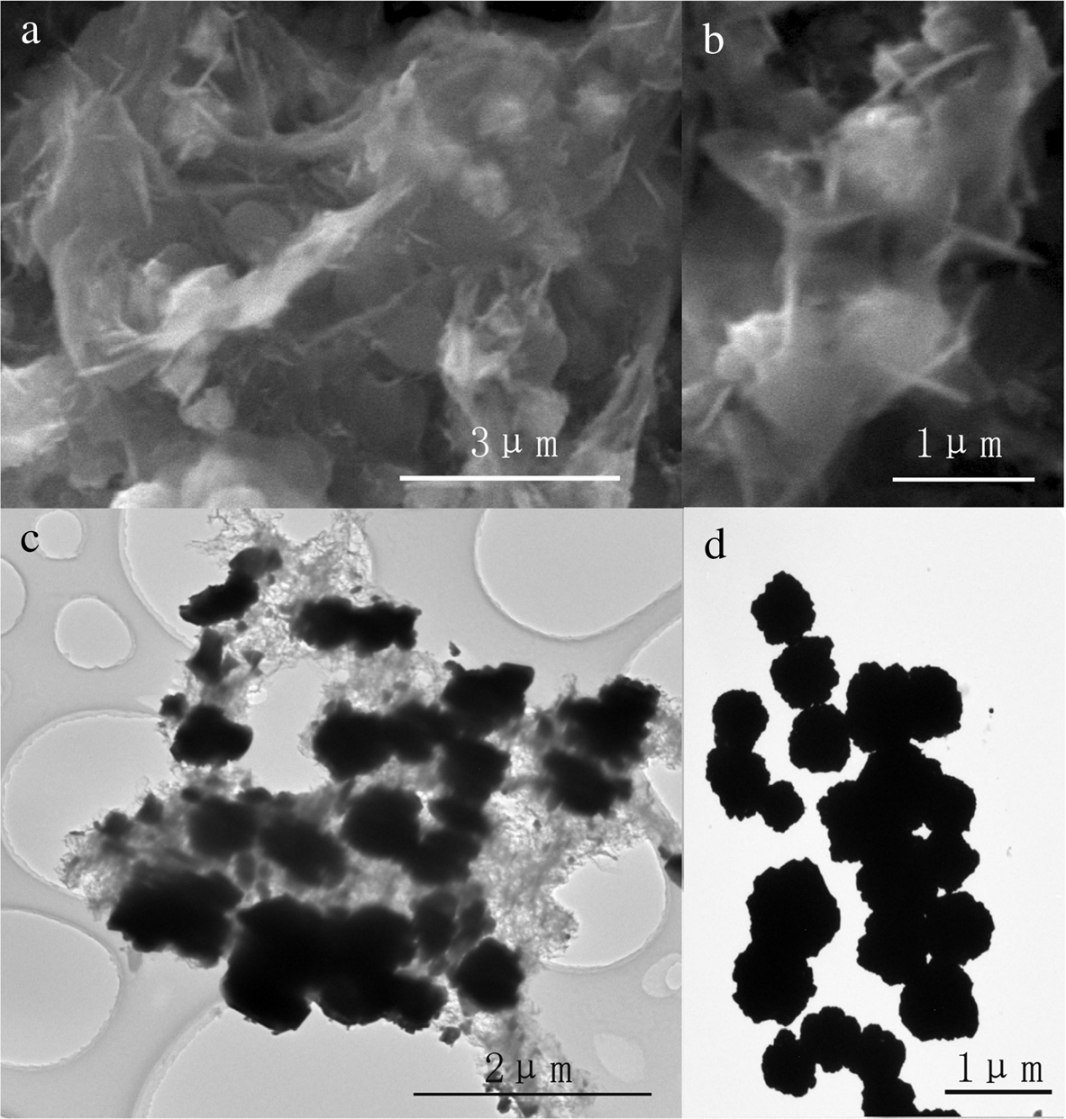
**SEM and TEM images of G/M-CdS composites and pure CdS MPs.** Typical SEM images of as-prepared G/M-CdS composites **(a, b)** and TEM images of G/M-CdS **(c)** and pure CdS MPs **(d)**.

The morphologies of the as-prepared G/M-CdS composites were characterized by SEM and TEM. As shown in Figure 2a, the CdS particles are well anchored on the graphene sheets. The partially enlarged image in Figure 2b reveals that the CdS MPs were coated by graphene sheet clearly. Further evidence for the attachment of CdS MPs onto the graphene is provided by TEM. Figure 2c shows a typical graphene nanosheet decorated by CdS MPs. It can be clearly observed that graphene nanosheets are hybridized with CdS MPs which are anchored on the graphene uniformly. Except for the CdS MPs decorating the graphene nanosheet, no other particles can be observed, which indicates the good combination of graphene and CdS MPs. The measurement of the size distribution shows that the CdS MPs in the hybrid have a relatively average diameter around 640 nm. For comparison, the TEM image of pure CdS MPs is shown in Figure 2d, which gives similar size distribution with that of CdS MPs in the hybrid.

**Figure 3:**
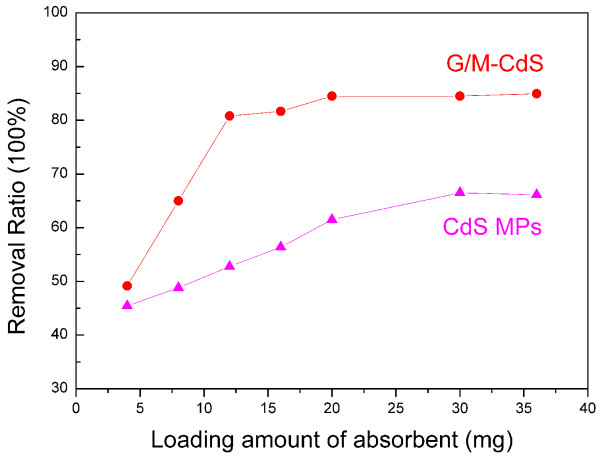
Adsorption capacity of Rh.B onto G/M-CdS composites and pure CdS MPs with different loading amount.

The adsorption of Rh.B was enhanced gradually before 150 min in the dark, when the adsorption-desorption equilibrium was reached. Figure 3 shows the adsorption capacity of Rh.B onto G/M-CdS composites and pure CdS MPs with different loading amount recorded at 150 min. The removal ratio of Rh.B increases with the increasing loading amount of G/M-CdS. The removal ratio of the dye is increased from 49.1% to 84.5% when the loading amount increases from 4 to 36 mg, which is higher than that of pure CdS MPs. The higher extraction efficiency of G/M-CdS could be attributed to the large surface area and high adsorption ability of the graphene. The mechanism of the G/CdS adsorption toward the organic dye may be derived from two reasons. One reason might be based on van der Waals interactions occurring between the hexagonally arrayed carbon atoms in the graphite sheet of G/CdS and the aromatic backbones of the dye. The second reason might be due to the strong *π*-stacking interaction between the benzene ring of the dye and the large delocalized *π*-electron system of the G [37]. It can be seen that the removal ratio gets to saturation when the loading amount of G/M-CdS is more than 20 mg.

**Figure 4:**
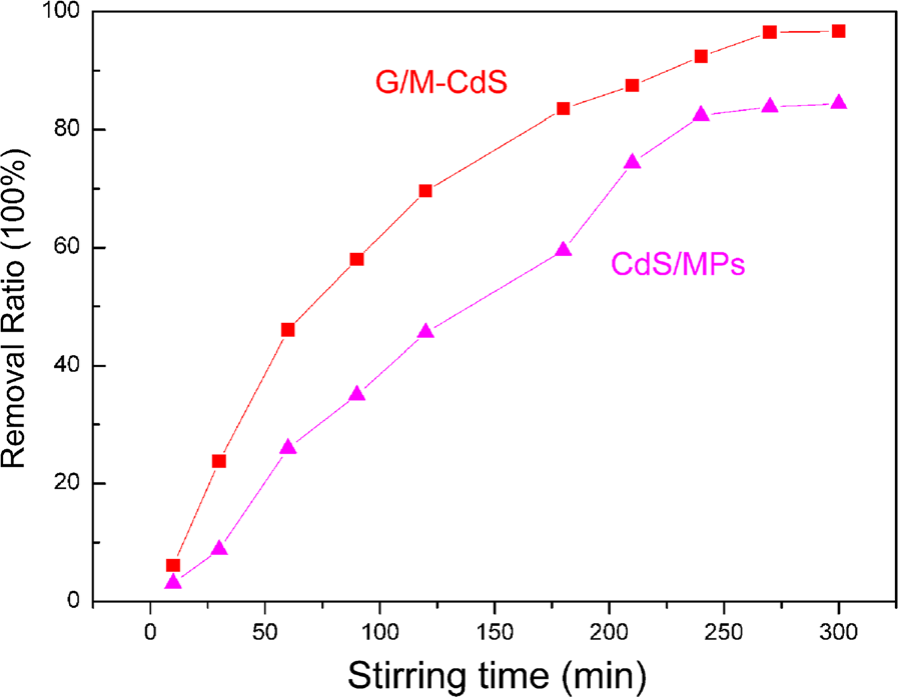
**Removal ratio of G/M-CdS and pure CdS MPs with increasing stirring time under visible-light irradiation.** The loading amount of both materials is 20 mg.

**Figure 5:**
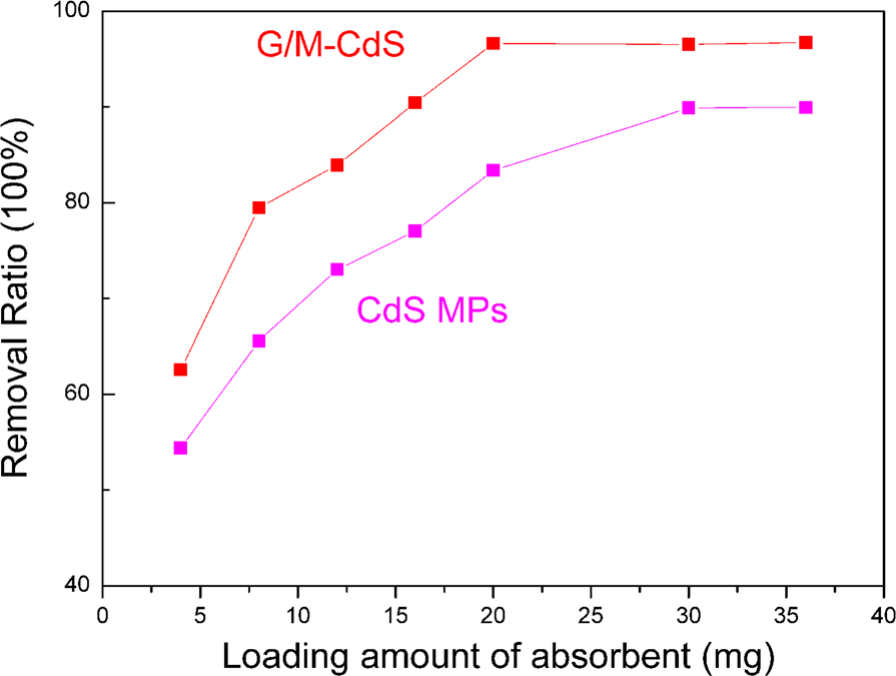
Removal ratio of G/M-CdS and pure CdS MPs with increasing loading amount under visible-light irradiation.

The photocatalytic performance of the G/M-CdS composites in terms of photodegradation of Rh.B molecules under visible-light irradiation was investigated. Figure 4 describes the removed Rh.B amount as a function of irradiation time. The loading amounts of G/M-CdS and CdS MPs are both 20 mg. When using G/M-CdS photocatalysts, the photodegradation rates of Rh.B had reached 69.5% after irradiating for 120 min. After the illumination time was extended to 270 min, 96.6% of Rh.B was decomposed. For pure CdS MPs, the photodegradation rate of Rh.B was 83.8% after 270 min visible light irradiation. It is seen that the photocatalytic activity of the G/M-CdS is better than that of pure CdS MPs. Figure 5 shows the removal ratio of Rh.B with increasing loading amount of absorbent under visible-light irradiation recorded at 270 min. For the G/M-CdS, the photodegradation ratio of Rh.B keep increasing from 4 to 20 mg, after which it keeps constant; for CdS MPs, the photodegradation ratio of Rh.B gets to maximum at 30 mg. This is consistent with the result of adsorption-desorption equilibrium experiment, and the suitable loading amount of the G/M-CdS composites should be 20 mg in this work.

**Figure 6:**
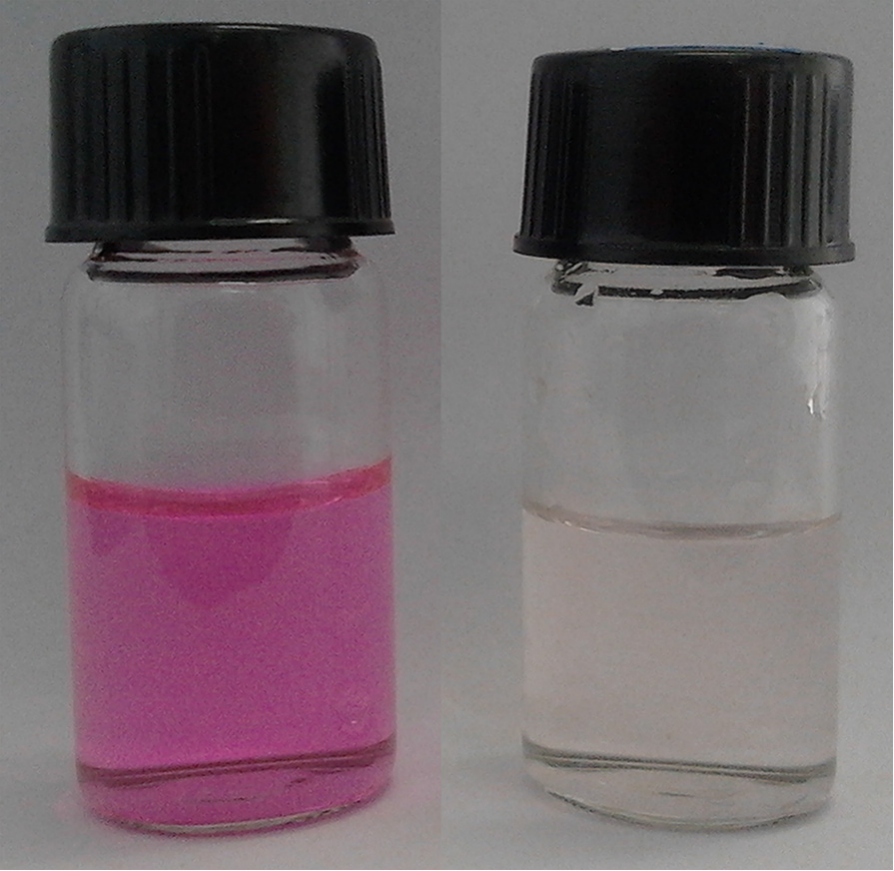
Rh.B solution (0.01 mg/mL, left) before and after separation of G/M-CdS adsorbent after photodegradation (right).

**Figure 7:**
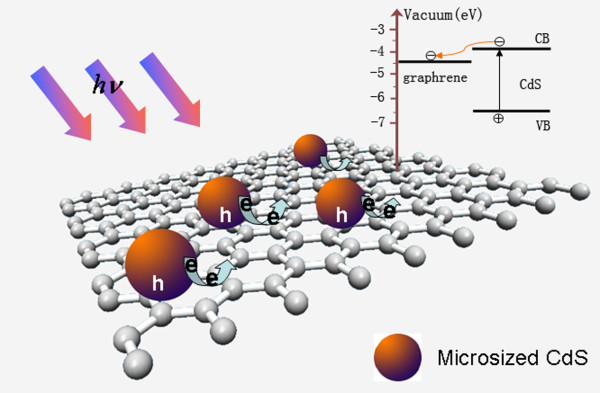
Illustration of charge separation and transfer in G/M-CdS system.

The adsorption characteristics of the G/M-CdS composites are displayed in Figure 6. It can be seen that, after stirring the mixture of the G/M-CdS composites and Rh.B aqueous solution (Figure 6, left) under visible-light irradiation for 270 min, the supernatant turned nearly colorless (Figure 6, right). This proved that the G/M-CdS composites possessed the properties of adsorption capacity and photodegradation. We would like to attribute the high efficient photodegradation activity to the electron transfer from CdS to graphene. As shown in Figure 7, CdS can be excited by UV light to generate electrons and holes. Then, the photogenerated electrons transfer to graphene while holes are left behind in CdS since the conduction band of CdS is more negative. This electron transfer route reduces the possibility of recombination of electron-hole pairs and prolongs the lifetime of charge carriers. In other words, the transfer of photoexcited electrons from CdS to graphene facilitates the charge separation, producing more –OH responsible for photodegradation of Rh.B. Previous reports on graphene-CdS composites as the adsorbent for the extraction of organic pollutants were mainly focused on nanoscaled CdS particles. Herein, the adsorption performance and photocatalytic activity of the large-sized CdS/G composite with approximately 0.64 μm CdS particles were investigated, and the results exhibited that the current composites possess comparable purification ability of waste water with that of nanoscaled CdS/graphene composites. The accurate decision of size effect of large CdS particles needs further investigation, which is a subject of our future research.

## Conclusions

In summary, we have successfully prepared G/M-CdS composites via an effective solvothermal method. Their ability of extraction of dye from aqueous solution was examined using Rh.B as adsorbate. The photocatalytic activity measurements demonstrate that the G/M-CdS photocatalysts show superior photoactivity in degradation of Rh.B under visible light irradiation. The present work opens up a new avenue to preparing G-based composite materials and provides new insights into the photocatalytic degradation of dyes under visible light irradiation.

## Abbreviations

G/M-CdS: CdS microparticles-graphene composites; GO: graphite oxide; MPs: microparticles; Rh.B: Rhodamine B; TEM: transmission electron microscopy; XRD: X-ray diffraction.

## Competing interests

The authors declare that they have no competing interests.

## Authors’ contributions

WL and CJ conceived the idea and carried out the experiments. CJ, WY, and DF, participated in the preparation of the samples. PZ, CJ, WY, DF, YY, and XG took part in the experiments and the discussion of the results. WL drafted the manuscript. All authors read and approved the final manuscript.
